# Paclitaxel-Induced Src Activation Is Inhibited by Dasatinib Treatment, Independently of Cancer Stem Cell Properties, in a Mouse Model of Ovarian Cancer

**DOI:** 10.3390/cancers11020243

**Published:** 2019-02-19

**Authors:** Elif Kadife, Emily Chan, Rodney Luwor, George Kannourakis, Jock Findlay, Nuzhat Ahmed

**Affiliations:** 1 Fiona Elsey Cancer Research Institute, Ballarat, VIC 3350, Australia; Elif@fecri.org.au (E.K.); George@fecri.org.au (G.K.); 2 Department of Obstetrics and Gynaecology, University of Melbourne, Melbourne, VIC 3052, Australia; emily.ern@gmail.com (E.C.); jock.findlay@hudson.org.au (J.F.); 3 Department of Surgery, Royal Melbourne Hospital, University of Melbourne, Melbourne, VIC 3052, Australia; rluwor@unimelb.edu.au; 4 School of Health and Life Science, Federation University Australia, Ballarat, VIC 3010, Australia; 5 Centre for Reproductive Health, The Hudson Institute of Medical Research, Clayton, VIC 3168, Australia

**Keywords:** ovarian carcinoma, ascites, chemoresistance, chemotherapy, Dasatinib, paclitaxel, cancer stem cells

## Abstract

Approximately seventy percent of ovarian cancer patients succumb to the disease within the first 5 years of diagnosis, even after successful surgery and effective chemotherapy treatment. A small subset of chemotherapy resistant cancer stem cells (CSCs) cause relapse of ovarian cancers. This study investigated the association between paclitaxel-mediated Src activation (p-Src) and CSC populations in driving ovarian cancer progression. We demonstrate that patients with high-stage serous ovarian carcinomas have significantly elevated levels of p-Src, compared to patient with low-stage and benign ovarian tumours. Additionally, p-Src was significantly enhanced in ascites-derived tumour cells obtained from recurrent patients, compared to chemonaïve patients. Paclitaxel treatment increased Src activation in ovarian cancer cells, causing enrichment of CSC marker expression in the surviving cells in vitro and in xenografts of nude mice. Dasatinib in combination with paclitaxel significantly suppressed p-Src in ovarian cancer cell lines and xenografts but had no effect on the expression of CSC markers. However, combination of paclitaxel and Dasatinib showed lower trend in invasion in liver and pancreas, compared to paclitaxel-only treatment. The tumours treated with combination therapy also had significantly lower infiltration of mononuclear cells. Robust recurrent tumour growth was observed in all mice groups after termination of treatments. The above results suggest that Dasatinib-mediated inhibition of p-Src may not be crucial for paclitaxel-induced CSC-mediated recurrence in ovarian cancer.

## 1. Introduction

Every twelve hours one woman dies of ovarian cancer worldwide. The high mortality rate in ovarian cancer patients results from the diagnosis of the disease at an advanced-stage when the cancer has already spread into the peritoneal cavity [1]. The management of ovarian cancer patients after debulking surgery involves systemic administration of platinum (cisplatin/carboplatin) and taxane-based (paclitaxel) drugs [2]. This treatment regimen results in a significant reduction of tumour burden due to substantial cancer cell death. Hence, approximately 80% of ovarian cancer patients respond to the initial standard treatment regimen and enjoy a short-lived period of remission with asymptomatic minimal disease [3]. However, the microscopic residual disease persisting after the first line chemotherapy usually gives rise to consecutive episodes of recurrent disease and eventual death. Disappointingly, a five-year survival period of ovarian cancer patients has remained unchanged and as low as ~30–40% for the last forty years [3]. Hence, there is an urgent need to identify and target mechanisms, which allow surviving residual cancer cells to overcome the cytotoxic effects of the chemotherapy and propagate within the changed tumour microenvironment.

Due to the anatomical positioning of the ovaries and minimal physical barriers, ovarian cancer cells readily metastasize and colonise within the parietal and visceral peritoneum, including omentum, diaphragm and small bowel mesentery [4]. This event is further augmented by the dedifferentiation of cancer cells in the progressing tumour microenvironment, particularly after the exposure of cytotoxic chemotherapy that imposes an inflammatory microenvironment [5]. The residual cells, after chemotherapy treatment, emerge with cancer stem cell-like (CSC) characteristics and phenotypic changes allowing them to propagate rapidly, overcoming apoptotic signals and immune regulation [5]. Furthermore, in the tumour microenvironment the stem-cell like cancer cells use self-modulated vessels that support angiogenesis, further aiding tumour growth and cancer metastasis [6,7]. Hence, the changes in the chemotherapy triggered microenvironment imposes alteration in signalling pathways in chemotherapy resistant stem-cell like cancer cells, which ultimately drives the disease into relapse and further consecutive episodes of recurrences [8,9].

Enhanced expression of Src kinase in cancers has been shown to deregulate cell cycle and cancer cell survival [10,11]. In ovarian cancer, Src is expressed at late stages and in poorly differentiated cancer cell lines [12,13]. In a panel of 119 ovarian tumours, patients identified as having dysregulation in Src gene were found to have worst prognosis. Poor prognostic outcomes associated with increased Src expression are attributed to its correlation with paclitaxel resistance. Inhibition of Src has been shown to increase the sensitivity of ovarian cancer cells to paclitaxel treatment [13].

Recognising the clinical potential of Src targeting in improving disease outcomes, multi-kinase inhibitor Dasatinib has been used to treat several malignancies in preclinical studies and clinical trials. The use of Dasatinib was approved in 2006 by the Food and Drug Administration (FDA) for the treatment of imatinib-resistant or intolerant adult chronic myeloid leukemia, Philadelphia chromosome-positive acute myeloid or lymphoblastic leukemia [14]. Alongside the Src family kinases (Src, Lck, Yes, and Fyn), this inhibitor targets BCR-ABL protein, stem cell factor receptor (c-Kit), EPH receptor A2, and platelet-derived growth factor receptor (PDGFR)-β at nanomolar concentrations [15]. Screening of 34 human ovarian cancer cell lines revealed that high expression of Yes, Lyn, Eph2A, caveolin-1 and 2, moesin, annexin-1, and uPA, and low expression of insulin-like growth factor-binding protein 2 (IGFBP2) render the cells sensitive to Dasatinib treatment [16]. Additionally, co-treating ovarian cancer cells with Dasatinib renders these cells sensitive to the cytotoxic effect of paclitaxel or carboplatin based chemotherapeutic drugs [13,17].

The aim of this study was to evaluate the effect of Dasatinib on paclitaxel-induced CSC properties in ovarian cancer cell lines in vitro and in an in vivo mouse model. The main aim was to evaluate if treatment with Dasatinib in combination with paclitaxel may affect tumour burden, peritoneal dissemination and disease-free remission period in a mouse model. We chose two ovarian cancer cell lines representative of high-grade serous (HEY) and clear cell (TOV-21G) ovarian carcinomas to determine the in vitro effect of paclitaxel with or without Dasatinib treatment. HEY cell line was used to determine in the in vivo effect of paclitaxel with or without Dasatinib. For the first time, this study provides insight on the Src-mediated survival aspects of chemotherapy-treated residual ovarian CSCs, which trigger a recurrent disease. We demonstrate that, in mice Dasatinib in combination with paclitaxel had no significant effect in reducing tumour burden over paclitaxel-alone treatment. In addition, combination treatment had no effect on the CSC-like profile of mouse xenografts, nor did it prolong overall survival in tumour-bearing mice once the treatment with paclitaxel and Dasatinib was terminated. These results suggest that Dasatinib may be a good agent in reducing initial tumour burden but may not be an effective therapeutic for the better management of recurrent ovarian cancer patients long-term.

## 2. Results

### 2.1. Active Src Levels Are Increased in the Primary Ovarian Tumours of High-Stage Serous Ovarian Cancer Patients and in the Ascites-Derived Tumour Cells of Advanced-Stage Recurrent Patients

In this study, immunohistochemical analysis was performed to compare the expression of phospho (p)-Src and total (t)-Src between normal/benign tissues, low and high-stage ovarian tumours (Table 1). Eight ovarian tissues samples were obtained from non-ovarian carcinoma patients, which consisted of two normal ovarian tissue samples from patients undergoing hysterectomy or bilateral salpingo-oophorectomy due to pre-diagnosed medical conditions. Six benign ovarian tumour samples were obtained from patients undergoing hysterectomy due to the presentation of cysts. Ovarian tissues from serous ovarian cancer patients consisted of three low-stages (FIGO stage I) and ten advanced-stage (FIGO stages III and IV) samples, collected at the time of primary ovarian cancer surgery (patient details are summarized in Table 1).

**Figure 1:**
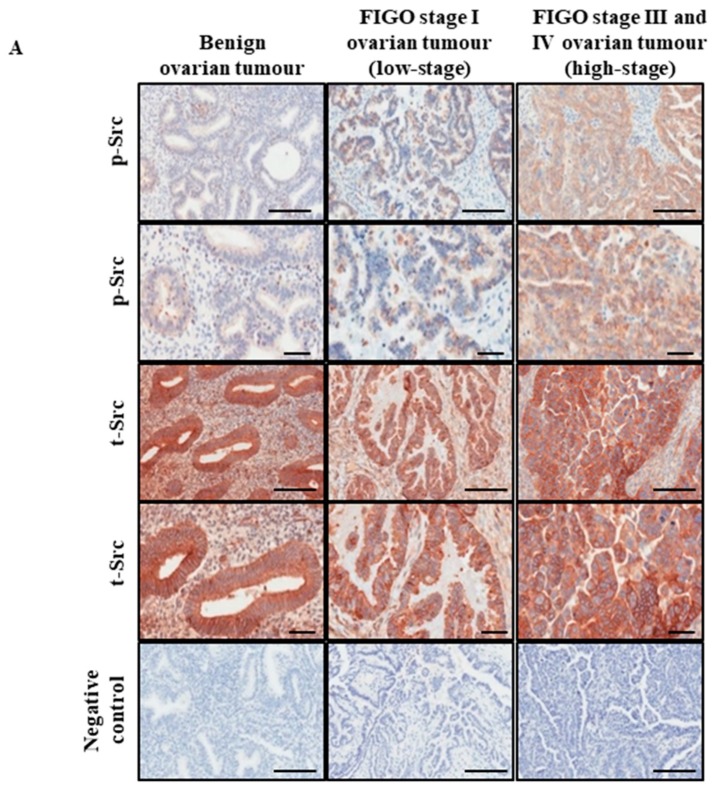

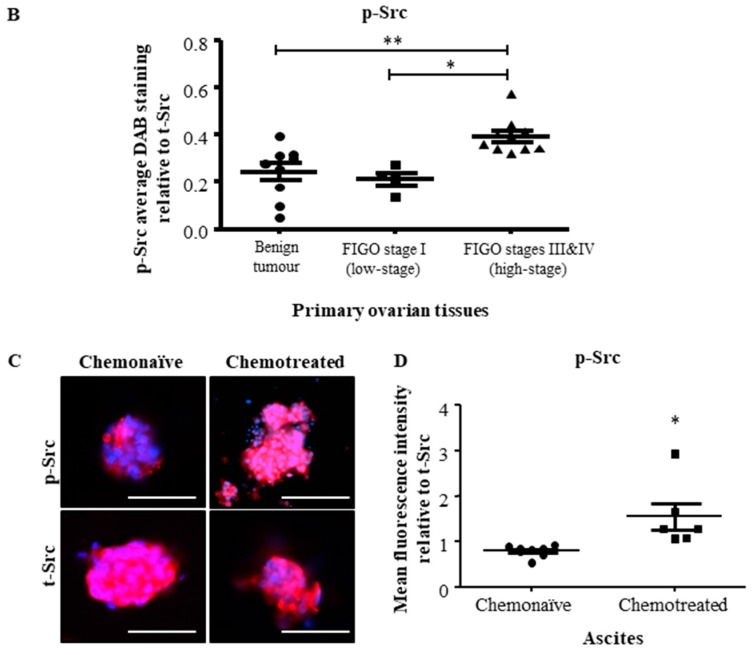
Late-stage and chemotherapy treated ovarian cancer patient samples have greater level of p-Src than early-stage and chemonaïve patients. (**A**) Representative images of p-Src and t-Src staining in primary ovarian benign tumours, FIGO stage I (low-stage) and III/IV (high-stage) serous ovarian cancer patients. Magnification 200× scale bar = 200 µM and 400× scale bar = 60 µM. (**B**) Quantification of p-Src and t-Src DAB staining was performed using Fiji software. Results are displayed as overall average DAB reading of p-Src relative to t-Src of the same samples ± SEM. (**C**) Expression and localization of the Src pathway activation was evaluated by immunofluorescence in non-adherent tumour cells derived from the ascites of chemonaïve and chemotreated recurrent serous ovarian cancer patients. Staining was visualized using the secondary Alexa 590 (red) and nuclei were detected by DAPI (blue) staining. Images are representative of *n* = 8 chemonaïve and *n* = 6 chemo-treated ascites-derived cells. Magnification 400× scale bar = 250 µM. (**D**) Quantification of t-Src and p-Src fluorescent intensities was determined using Fiji software. Results are displayed as average fluorescent intensity value of p-Src relative to t-Src of the same ascites sample ± SEM. Significance is indicated by * *p* < 0.05, ** *p* < 0.01.

The expression of p-Src was significantly greater in high-stage serous ovarian tumours, compared to low-stage and benign ovarian tumours (Figure 1A,B). The level of expression and activation of Src was assessed by immunofluorescence in non-adherent cell populations derived from ascites of unmatched advanced-stage recurrent serous ovarian cancer patients, previously treated with chemotherapy (*n* = 6) and advanced-stage chemonaïve serous ovarian cancer patients (*n* = 8) (Table 2). Activated p-Src protein localized more in the nucleus in ascites-derived cells from recurrent patients, compared to those who were chemonaïve (Figure 1C). The mean fluorescent intensity of p-Src relative to t-Src was approximately 2-folds higher in chemotherapy-treated recurrent patients, compared to chemonaïve patients (Figure 1D).

### 2.2. Src Activation Was Transiently Induced in Ovarian Cancer Cell Lines by Paclitaxel Treatment

To determine if the activation of Src can be upregulated in ovarian cancer cells in response to chemotherapy and to mimic the profile seen in the ascites-derived tumour cells of chemotherapy treated patients, the HEY ovarian cancer cell line was subjected to IC50 concentration of paclitaxel (0.05 µg/mL) for 6, 24 and 72 h (Supplementary Figure S1). Src expression was assessed by immunofluorescence and western blot analysis.

**Figure 2:**
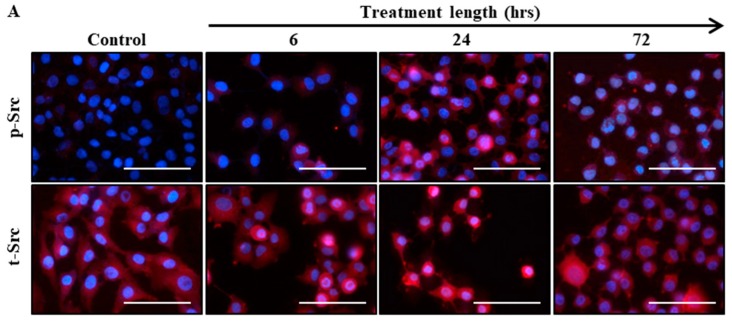

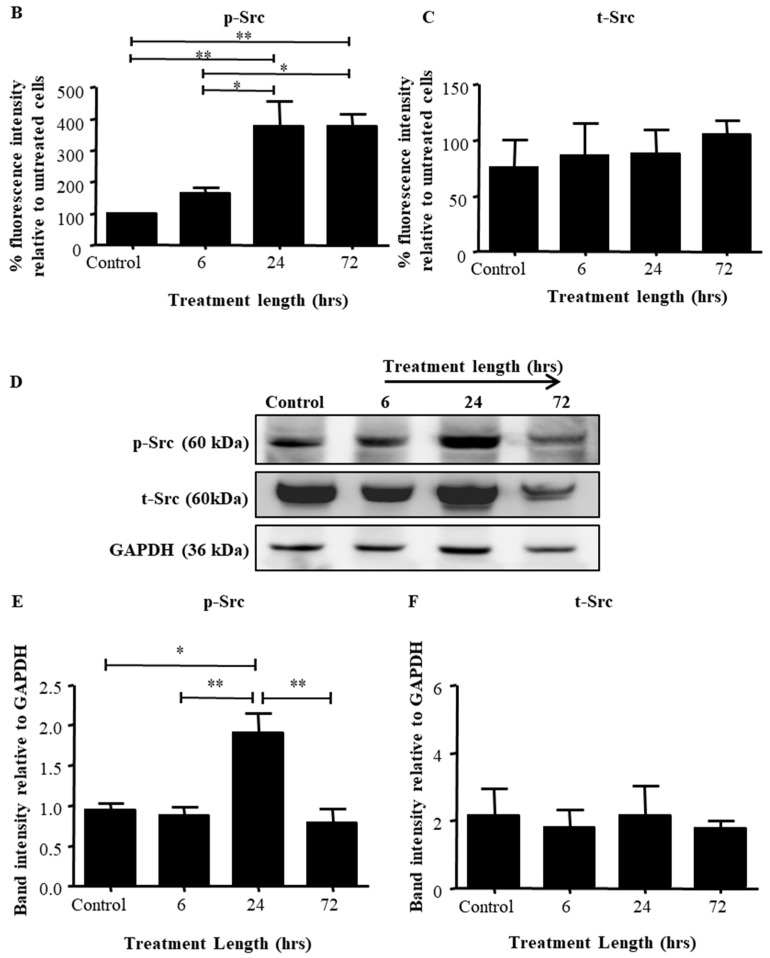
Exposure of HEY cells to paclitaxel enhances phosphorylation of Src in a time dependent manner. (**A**) The expression of p-Src and t-Src was assessed by immunofluorescence in untreated and paclitaxel (0.05 µg/mL) treated cells following 6, 24 or 72 h of incubation. Staining was visualized using the secondary Alexa 590 (red) fluorescent-labelled antibody, and nuclei were detected by DAPI (blue) staining. Magnification 400× scale bar = 250 µm. Quantification of (**B**) p-Src and (**C**) t-Src fluorescent intensities were conducted using Fiji software. Results are displayed as the percentage of the average fluorescent intensity relative to untreated cells ± SEM (*n* = 3/group). (**D**) Total cell lysates of HEY cells were collected at 6, 24 and 72 h after paclitaxel treatment and were subjected to immunoblot analysis using antibodies specific for p- or t-Src or GAPDH. Images are representative of three independent experiments. Densitometry analysis of (**E**) p-Src and (**F**) t-Src protein expressions. The values represent the relative mean of band intensity normalized to GAPDH loading control ± SEM. Significance is indicated by * *p* < 0.05, ** *p* < 0.01.

Immunofluorescent studies revealed that there was a nuclear translocation of p-Src from cytoplasm starting at 6 h (Figure 2A). This was more evident at 24 and 72 h of paclitaxel treatment (Figure 2B). However, the cytosolic expression of t-Src remained unchanged across all groups and time points (Figure 2C).

Western blot analysis showed that in HEY cells treated with paclitaxel, p-Src protein levels were significantly higher at 24 h compared to control, 6 and 72 h treatments (Figure 2D). The expression of p-Src at 6 and 72 h after paclitaxel treatment remained similar to the untreated cells (Figure 2E). T-Src expression remained unchanged between all groups (Figure 2F).

The patterns of p-Src expression in response to paclitaxel in TOV-21G cells showed Src activation within 24 h by immunofluorescence which diminished at 72 h (Supplementary Figure S2A,B). However, western blot analysis revealed sustenance of that activation by the 72 h time point (Supplementary Figure S2D,E). T-Src expression remained unchanged between all groups (Supplementary Figure S2C,F).

### 2.3. The Addition of Dasatinib Suppressed Paclitaxel-Induced Src Activation in Ovarian Cancer Cells

Immunofluorescence was used to investigate the effect of Dasatinib on inhibiting Src activation in HEY cells, when given alone (10 µM) and in combination with paclitaxel (0.05 µg/mL) (Supplementary Figures S1 and S3).

**Figure 3:**
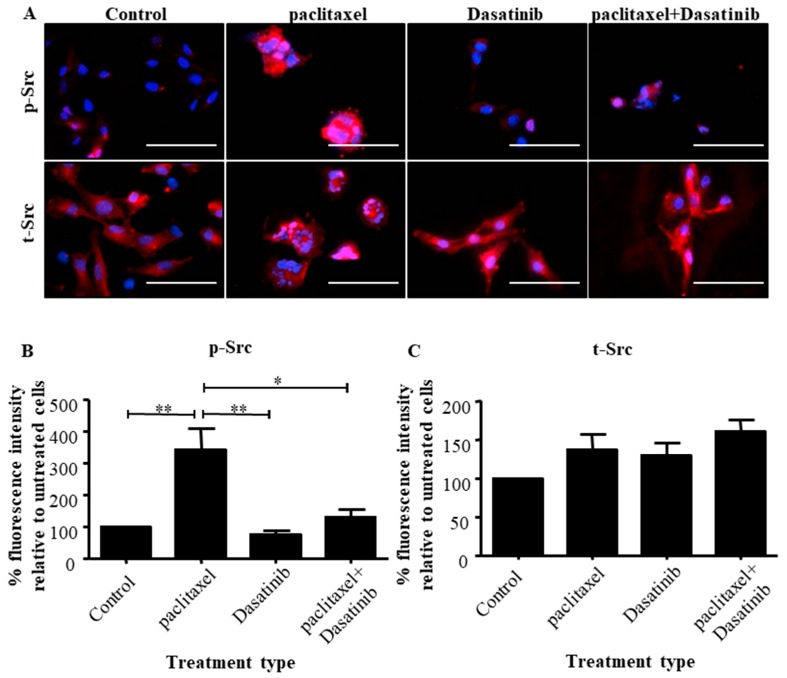
Dasatinib inhibits paclitaxel-induced Src activation in HEY cells. (**A**) Immunofluorescent visualization of expression and localization of the p- or t-Src proteins in untreated HEY cells or following a 24 h treatment with paclitaxel (0.05 µg/mL), Dasatinib (10 µM) or a combination of both. Staining was visualized using the secondary Alexa 590 (red) fluorescent-labelled antibodies and nuclei were detected by DAPI (blue) staining. Images are representative of three independent experiments. Magnification 400× scale bar = 250 µm. Quantification of (**B**) p-Src and (**C**) t-Src fluorescent intensities were determined using Fiji software. Results are expressed as the percentage of the average fluorescent intensity value relative to untreated cells ± SEM (*n* = 3/group). Significance between the groups and is indicated by * *p* < 0.05, ** *p* < 0.01.

Enhanced intensity of nuclear localisation of p-Src was evident in paclitaxel treated cells compared to control untreated cells (Figure 3A). Quantification of the fluorescent intensity of p-Src proteins showed significant greater levels of activated protein in the paclitaxel treated cells, compared to the untreated group (Figure 3B). Dasatinib treated cells had a similar intensity of p-Src as untreated cells, which was significantly lower than the cells treated with paclitaxel. In addition, the combination of Dasatinib with paclitaxel significantly inhibited p-Src compared to paclitaxel-only treated groups (Figure 3A,B). There was no significant difference in the expression of t-Src between control and treatment groups (Figure 3C).

These results were consistent in the ovarian cancer TOV-21G cell line which showed a significant elevation of p-Src in response to paclitaxel treatment which was abolished by Dasatinib treatment (Supplementary Figure S4A,B). No significant difference in the expression of t-Src and between control and treatment groups was also observed (Supplementary Figure S4C).

### 2.4. Dasatinib Reduced Some Paclitaxel-Induced CSC-Like Marker Expression in Ovarian Cancer Cells

The effect of Dasatinib on paclitaxel-induced CSC-like marker expression of ovarian cancer cells was analysed using flow cytometry. The expression of the markers EpCAM, CD44, CD133 and SSEA-4 were analysed by subjecting HEY cells to IC50 concentrations of paclitaxel (0.05 µg/mL), Dasatinib (10 µM) and a combination of both.

**Figure 4:**
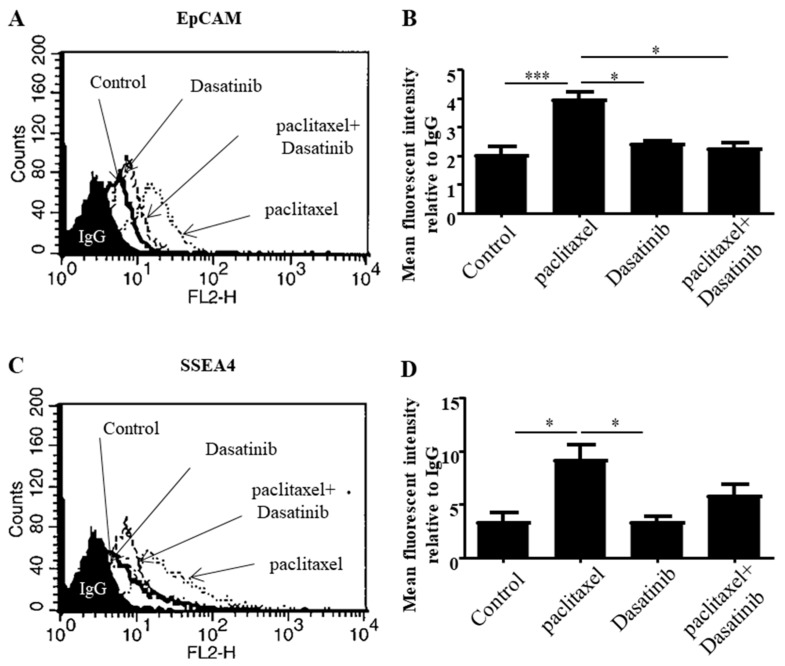

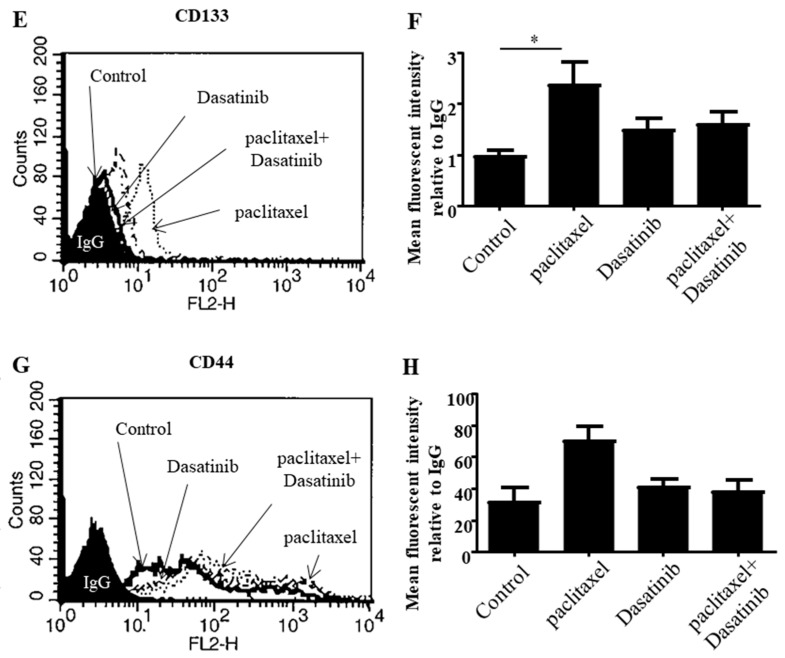
CSC-like marker expression is reduced with Dasatinib treatment. Surface expression of (**A**,**B**) EpCAM, (**C**,**D**) SSEA-4, (**E**,**F**) CD133 and (**G**,**H**) CD44 in ovarian cancer HEY cells, following a 24 h treatment with paclitaxel, Dasatinib or a combination of both. Histograms are representative of four independent experiments. Semi-quantitative analysis of the arbitrary fluorescent expressions of (**B**) EpCAM, (**D**) SSEA-4, (**F**) CD133 and (**H**) CD44 was determined by using Cell Quest^TM^ software. The values represent the mean of fluorescent intensity relative to the mean of control IgG ± SEM (*n* = 4). Significance is indicated by * *p* < 0.05, *** *p* < 0.001.

Paclitaxel significantly enhanced the expression of EpCAM in HEY ovarian cancer cell line and it was significantly suppressed by the addition of Dasatinib (Figure 4A,B). SSEA-4 and CD133 CSC-like markers were significantly upregulated by paclitaxel treatment. Even though Dasatinib had a decreasing effect on the paclitaxel-induced expression of these markers it was not significant (Figure 4C–F). Furthermore, there was an insignificant but increasing trend in the expression of CD44 in response to paclitaxel treatment. Dasatinib slightly decreased this effect on the expression of CD44 (Figure 4G,H).

On the other hand, paclitaxel induced significant upregulation in the expression of EpCAM, and CD133, which was inhibited by Dasatinib in TOV-21G cell line (Supplementary Figure S5A–H). The expression of SSEA-4 and CD44 had an increasing trend with paclitaxel treatment in TOV-21G cells, which was decreased by Dasatinib without significance.

### 2.5. Combination Treatment Had no Significant Effect in Reducing Tumour Burden Over Paclitaxel but Inhibited Src Activation in HEY Derived Xenografts

**Figure 5:**
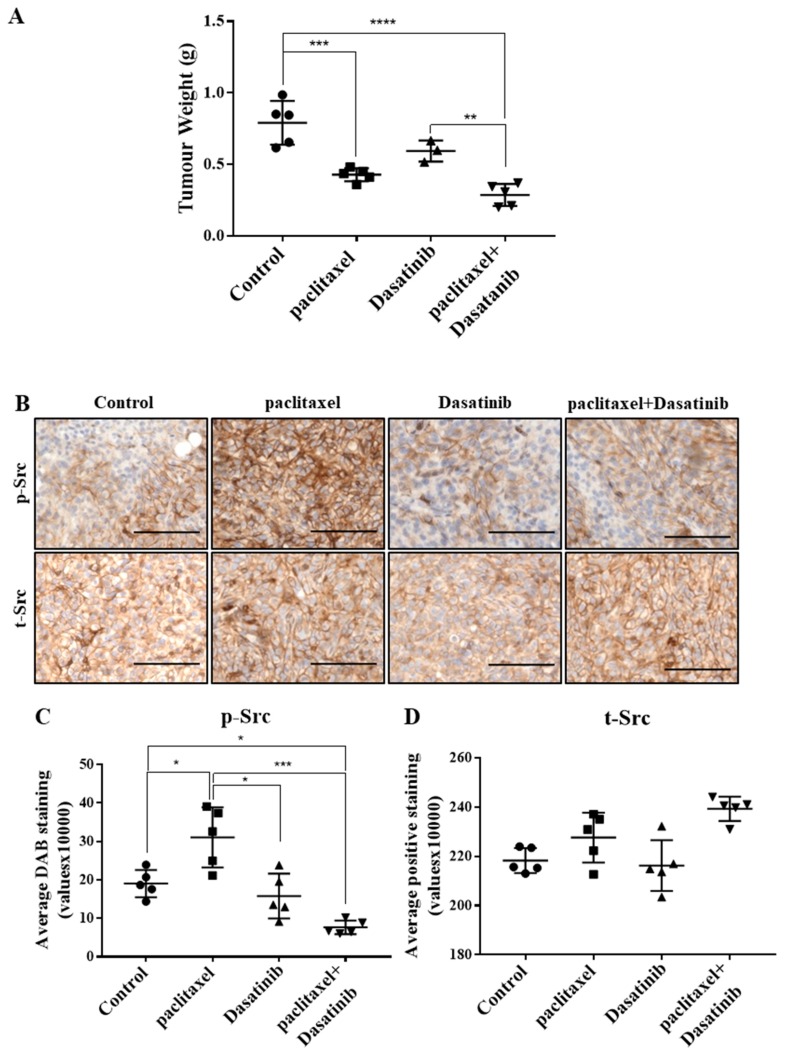
Combination of paclitaxel and Dasatinib treatment regimen in mice reduces tumour burden compared to control group but not to paclitaxel treated group and inhibits the paclitaxel-induced p-Src expression in tumours. (**A**) Tumour burden represented as collective tumour weights, 30 days post transplantation of cells and 12 days post treatment. (**B**) Representative images of p- or t-Src staining in HEY cell derived xenografts. Magnification 200× scale bar = 200 µM. Quantification of (**C**) p-Src and (**D**) t-Src, DAB staining was performed using Fiji software. Results are displayed as average DAB staining of the same samples (×10,000) ± SD. Significance is indicated by * *p* < 0.05, ** *p* < 0.01, *** *p* < 0.001, **** *p* < 0.0001.

To investigate the in vivo anti-tumourigenic effect following the administration of Dasatinib in conjunction with paclitaxel, mice were killed at the 30-day endpoint (12-day of treatment) and their tumour burden was measured. Similar to previously reported results [18], upon dissection, multiple tumour deposits were observed in the liver, pancreas and bowels and several smaller tumour nodules were found throughout the peritoneal cavity of control untreated mice. Mice treated with paclitaxel or Dasatinib developed smaller tumour nodules and fewer tumours were found from each mouse compared to control untreated mice. We compared the tumour burden between all mice in the study. Untreated control mice developed the largest tumour burden compared to all groups (Figure 5A). Significantly reduced tumour burden was observed in the group injected weekly with paclitaxel compared to control mice. Mice treated with the combination of paclitaxel and Dasatinib also had significantly smaller tumour burden compared to control animals, however, this was not significantly different compared to paclitaxel-alone treated mice. Daily Dasatinib treatment reduced tumour burden to similar levels observed in the paclitaxel treated mice, however, this was significantly larger than mice treated with combination of the two drugs. Paclitaxel, Dasatinib or a combination of both were well tolerated in mice without any major adverse effects. In line with in vitro results, the tumours generated with paclitaxel alone treatment had significantly higher p-Src protein, which was inhibited significantly by a combination of paclitaxel and Dasatinib treatments (Figure 5B,C). The p-Src levels of combination treated xenografts were significantly lower than control and paclitaxel-alone treated tumours (Figure 5C). The t-Src levels remained relatively unchanged between the experimental groups (Figure 5D).

### 2.6. Inhibition of p-Src by Dasatinib Had no Significant Effect on Paclitaxel-Induced Expression of CSC, Proliferation, Tumourigenicity or the Vascularization Markers

**Figure 6:**
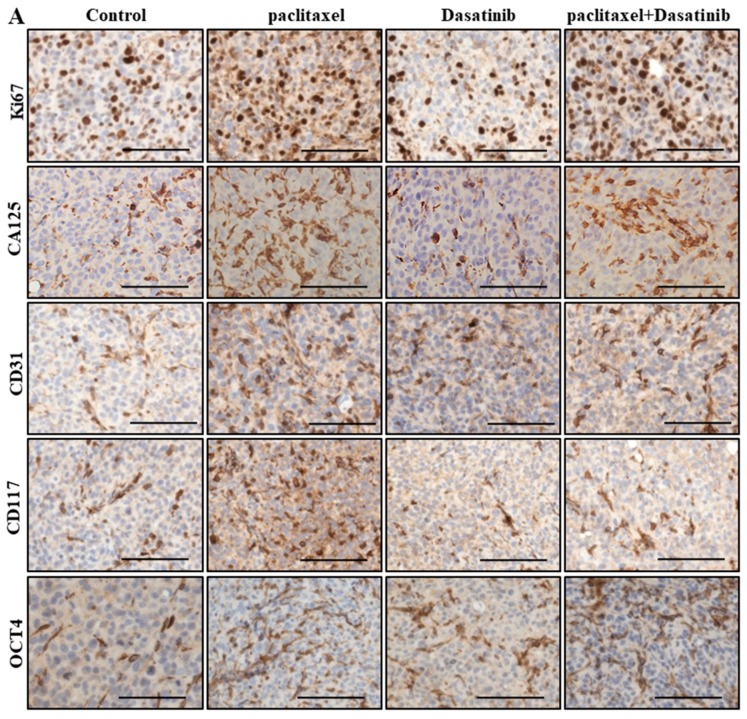

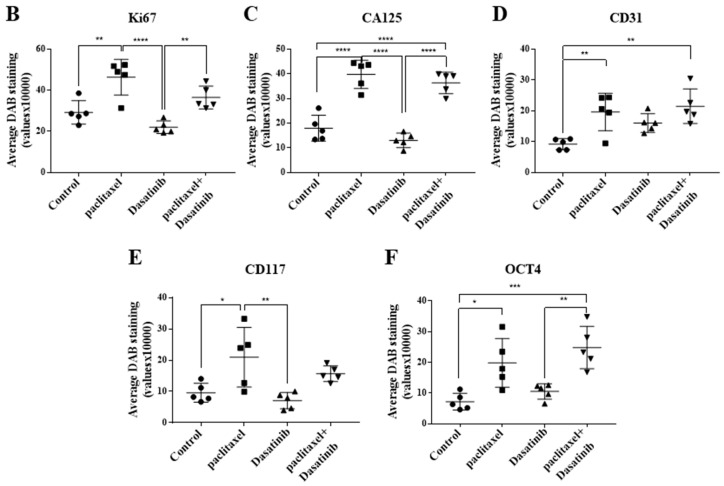
Dasatinib and paclitaxel combination treatment does not reduce paclitaxel-induced Ki67, CA125, CD31 or CSC marker expressions in ovarian xenografts. (**A**) Representative images of Ki67, CA125, CD31, CSC markers in HEY cell derived xenografts, post 12 days of treatment. Magnification 200× scale bar = 200 µM. Quantification of (**B**) Ki67, (**C**) CA125, (**D**) CD31, (**E**) CD117 and (**F**) OCT4 DAB staining was performed using Aperio software. Results are displayed as average DAB staining of the same samples (×10,000) ± SD. Significance is indicated by * *p* < 0.05, ** *p* < 0.01, *** *p* < 0.001, **** *p* < 0.0001.

Paclitaxel-treated tumours had a significantly greater percentage of Ki67 positive proliferating cells than untreated control xenografts (Figure 6A,B). Despite the significantly less Ki67 positivity in Dasatinib-alone treated tumours, the combined treatment group did not show a significant reduction in the Ki67 positive cells compared to the paclitaxel-treated group (Figure 6A,B). Combination treated tumours had significantly higher levels of Ki67 positivity than the Dasatinib alone treated groups.

CA125 is used as a predictive biomarker in ovarian cancer screening and monitoring. Treatment with paclitaxel significantly upregulated the expression of CA125 in HEY xenografts (Figure 6A–C). Treatment with Dasatinib had no effect on the expression of this marker compared to control untreated xenografts. Combined treatment with paclitaxel and Dasatinib did not reduce the expression of CA125 compared to paclitaxel treatment alone (Figure 6A,C).

Paclitaxel treatment of HEY cell-induced xenografts significantly enhanced the expression of CD31 in tumour cells, compared to untreated controls (Figure 6A,D). However, no difference in the expression of CD31 was observed in xenografts treated with a combination of paclitaxel and Dasatinib, compared to paclitaxel only treatment (Figure 6A,D). Dasatinib alone treated group had similar CD31 positive cells in comparison to other treatment groups (Figure 6A,D).

The tyrosine kinase oncoprotein, CD117 (c-Kit) is associated with chemotherapy resistance and can be used as a predictive CSC marker in ovarian cancer [19,20]. The paclitaxel-treated tumours expressed significantly greater level of this marker than untreated treated tumours (Figure 6A,E). However, combining Dasatinib with paclitaxel did not overcome this effect (Figure 6A,E).

Octamer-binding transcription factor 4 (OCT4) is an embryonic stem cell marker, the expression of which was upregulated in ovarian tumours to enhance cancer cell proliferation, migration and decreased cisplatin sensitivity [21]. Paclitaxel treatment significantly enhanced OCT4 expression levels in mouse xenografts (Figure 6A,F). Dasatinib only treatment did not induce a marked change in OCT4 expression compared to the untreated control group (Figure 6A,F). In paclitaxel and Dasatinib combined treatment groups, no change in the expression of OCT4 was observed compared to paclitaxel treated group (Figure 6A,F).

### 2.7. Tumours Treated with Dasatinib in Combination with Paclitaxel Had a Reduced Tendency to Invade Peritoneal Organs and Had Significantly Reduced Mononuclear Infiltrate

**Figure 7:**
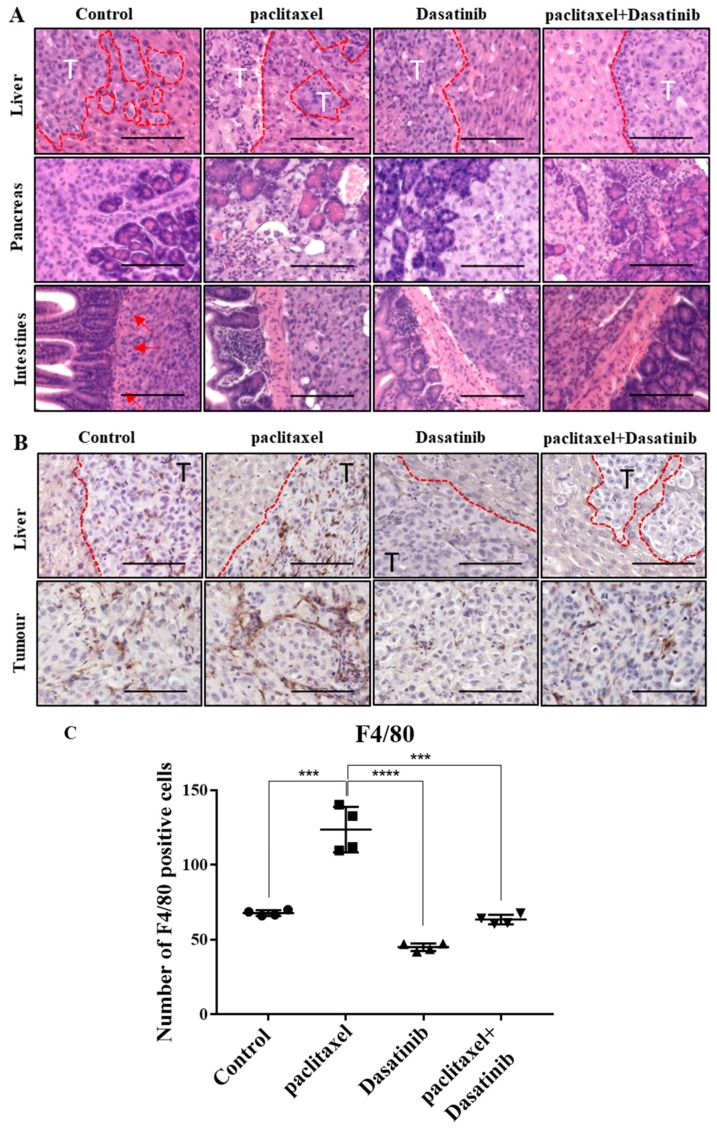
Dasatinib treatment reduces tumour invasion into organs and reduces infiltration of macrophages in tumours. (**A**) Histological analysis of liver, pancreas and intestines showing tumour attachment/invasion, post 12 days of treatment. Magnification 200× scale bar = 200 µM. (**B**) Immunohistochemical staining of F4/80 macrophage marker in liver lesions and omental tumours. Magnification 200× scale bar = 200 µM. (**C**) Quantification of F4/80 DAB staining in omental tumours using Fiji software. Results are displayed as number of positive cells ± SD. Significance between the groups and is indicated by ***p < 0.001, **** *p* < 0.0001.

Histological analysis of organs revealed that the induced tumours in the control group, were invasive and appeared to progress through adjacent peritoneal organs. In the liver, some lobes were completely replaced by tumours with further smaller invading frontal lesions (Figure 7A). Similarly, in the pancreas exocrine cells were segmented and in some regions were replaced by large tumour masses. Across all groups, the intestinal tract was the least invaded; however, some mice in the control group showed attachment and invasion into the muscular layers of the small intestines (Figure 7A). In the paclitaxel group, cells in the tumours appeared to be more disorganised than other groups. However, overall there was less penetration into organs, particularly the liver compared to the control group. On the other hand, the tumours treated with Dasatinib had less leading edges invading into the liver (Figure 7A). While some regions of the liver had high tumour presence, there were distinct borders between tumour and liver cells. In this group, there was minimal tumour attachment and invasion into small intestines. The combination treated tumours also revealed a reduced tendency for invasion into organs, particularly liver and pancreas.

In addition, the livers and tumours of mice treated with paclitaxel appeared necrotic with fibrosis and were surrounded by mononuclear cell infiltrates (F4/80 staining). Similar pattern of mononuclear cell infiltration was observed in tumours of control mice but to a less significant degree (Figure 7B). Mononuclear cell infiltration in Dasatinib and the combination treatment groups was significantly less than in the paclitaxel-treated group.

### 2.8. Termination of Paclitaxel, Dasatinib or a Combination of Both Treatments Resulted in Robust Tumour Development with no Change in Survival of Tumour Bearing Mice

**Figure 8:**
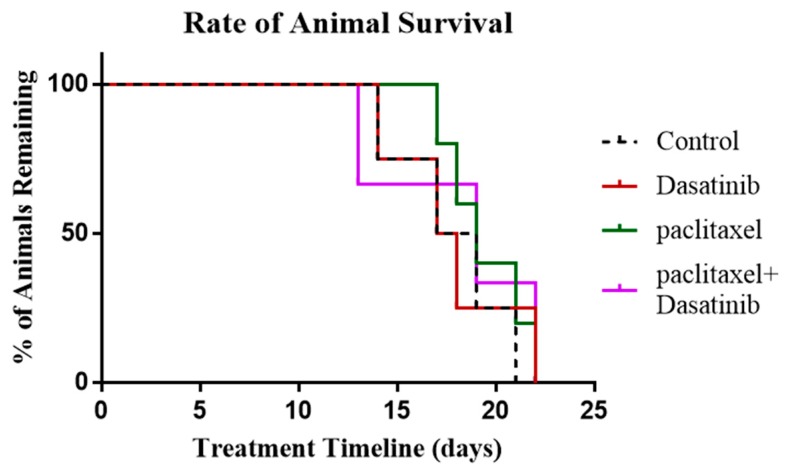
Survival analysis of paclitaxel, Dasatinib and combined paclitaxel and Dasatinib treated mice in recurrent phase. Kaplan-Meier survival analysis of control untreated mice (black dotted line), paclitaxel treated mice (green line), Dasatinib treated mice (red line) and paclitaxel and Dasatinib treated mice (purple line). Control mice were culled at the endpoint of experiment. The remaining groups of mice were monitored until they reached their individual endpoints.

In this experiment, mice were treated as described above until endpoint was reached by the control group, at which time treatment in all other groups was stopped. The survival period in mice were noted following termination of paclitaxel, Dasatinib or combination treatments, until the endpoint was reached by each individual animal. Overall, there was no significant difference in the survival of mice within groups treated with paclitaxel, Dasatinib and combination of the two (Figure 8).

## 3. Discussion

In this study, we report significantly high expression of phosphorylated Src in high-stage ovarian tumours compared to normal/benign and low-stage ovarian tumours. Src overexpression has been reported in various cancers, including ovarian cancer, during the progressive stages and is known to promote cancer cell survival and growth leading to poor survival in patients [22,23]. We also demonstrate that ascites-derived tumour cells, isolated from chemotherapy-treated recurrent ovarian cancer patients, have significantly high expression of activated Src compared to ascites-derived tumour cells of chemonaïve patients. This suggests that activated Src may have a potential role in facilitating chemoresistance which is associated with recurrence in ovarian cancer. Given that Src signalling has been previously implicated in promoting chemoresistance in ovarian cancer [13,23,24], in the present study we aimed to investigate the CSC-mediated anti-tumour activity of Dasatinib, alone and in combination with paclitaxel in vitro and in vivo.

Paclitaxel is a commonly used chemotherapeutic agent in ovarian cancer patients, however, some advanced-stage patients can present a high degree of paclitaxel resistance [25,26]. In this study, we show an upregulation of phophorylated Src in response to paclitaxel in two ovarian cancer cell lines in vitro. This upregulation was concomitant with a significant increase in the expression of CSC-like markers following paclitaxel treatment compared to untreated control cells. This suggests a potential association between the paclitaxel-induced activation of Src pathway and the emergence of CSC-like properties in ovarian cancer. We have previously reported emergence of CSCs in response to paclitaxel treatment in ovarian cancer cell lines and in vivo tumour xenografts induced either by intraperitoneal injection of ovarian cancer cells pre-treated with paclitaxel in vitro [27], or in response to paclitaxel treatment after ovarian cancer cells were injected intraperitoneal in mice [18,28]. These results support the theory that paclitaxel treatment of ovarian cancer cells provokes an escalation of CSC-like resistant progenies in residual ovarian tumour populations, which hypothetically may use the activated Src pathway to evade cytotoxic effects of the chemotherapy. In this context, targeting the CSC population through Src inhibition may provide an effective approach to overcome CSC-mediated chemoresistance. Recently, combination therapies that include a CSC-specific targeted agent and a non-CSCs-targeted agent such as chemotherapy, were suggested to be the most effective approach in the treatment of cancer [29].

Dasatinib is a dual Src/Abl kinase inhibitor that has been shown to possess anti-tumour activity in a number of solid tumours [30,31]. We studied the effect of Dasatinib in an intraperitoneal model of ovarian cancer metastasis, which mimics human progression of the disease. The tumour burden of untreated control mice was significantly higher than paclitaxel-alone and the combination of paclitaxel and Dasatinib treated groups. As expected, paclitaxel treatment reduced the burden by approximately half compared to the control group. However, Dasatinib in combination with paclitaxel had no additional effect in further decreasing the paclitaxel-reduced tumour burden. There were indications that paclitaxel-treated tumours had acquired an aggressive phenotype. Firstly, the cells comprising the tumours of paclitaxel treated mice were largely positive for p-Src. This upregulation of the active Src pathway may have benefited paclitaxel treated surviving cells in mouse tumours by allowing rapid renewal and survival, as indicated by the greater number of Ki67 positive cells found in these xenografts. Additionally, the paclitaxel treated tumours had high expression levels of CSC markers (CD117 and OCT4) and a significantly greater expression of tumourigenic CA125 and vascularisation marker CD31.

Dasatinib treatment alone appeared to impact tumour viability with reduced number of Ki67 positive proliferating cells, compared to paclitaxel treatment. However, combination treatment did not significantly suppress the paclitaxel-effect as Ki67 staining of these tumours remained significantly higher than control and Dasatinib-treated tumours. Dasatinib combined with paclitaxel had no significant effect on the paclitaxel-induced upregulation of CA125, CD117, OCT4 and CD31. Hence, Dasatinib in combination with paclitaxel did not significantly overcome the proliferative, tumourigenic, vasculogenic and CSC-like effect of paclitaxel in HEY cell induced ovarian tumours. However, mice administered with Dasatinib in combination with paclitaxel, lacked intensive invasion into pancreas and liver, major organs affected by ovarian tumour metastasis, suggesting that Dasatinib may hold some potential in targeting intra-peritoneal dissemination of ovarian cancer.

A cancer-rich inflamed microenvironment results in the recruitment of monocyte-macrophage lineage cells, which promote tumour growth and progression of cancers via release of cytokines and chemokines. The inflammatory environment also increases the frequency of regulatory T cells (Tregs) that assists immune evasion of cancer cells through T-cell suppression and activation [32]. High macrophage counts are associated with a poor prognosis in a number of cancers, including cervical, endometrial, prostate, lung, pancreatic cancers and glioblastomas [33]. Furthermore, macrophages have been shown to trigger therapy resistance by promoting signalling pathways in cancer cells [34]. In our study, we identified increased infiltration of F4/80 stained mouse macrophages in paclitaxel treated tumours compared to control tumours. As treatment with chemotherapy induces an inflammatory secretory DNA damage response in surviving cells, the infiltration of mouse macrophages in these tumours was not unexpected [35]. This infiltration of macrophages was reduced in Dasatinib-treated tumours. As such, inhibition of Src signalling in addition to suppression of infiltrating macrophages may be part of the mechanisms underlying Dasatinib-driven reduced tumour burden and invasion observed in our study [36]. Src family of kinases play a crucial role in myeloid cell recruitment and one of the side effects listed for Dasatinib in humans is the suppression of myeloid-derived cell infiltration [36]. However, immune cell infiltration and its potential in promoting or suppressing tumour progression is a subject of further investigation in future ovarian cancer studies.

Src kinases forms part of a large family of non-receptor tyrosine kinases that regulate a number of signaling pathways that impact on the behavior of tumour cells, including proliferation, survival, migration, invasion, and angiogenesis [37,38,39,40]. Src inhibitors (Dasatinib, saracatinib, bosutinib, etc) have been in clinical development, but their efficacy in clinical trials has not been adequate. In addition, as this vast family of tyrosine kinase regulate multiple functions in tumours, inhibition of specific members of Src family of kinases may not have the functional outcome due to compensatory increase in other members of the family [41]. Moreover, Src kinases play an important role in the development of host immune responses; such as the development and activation of T lymphocytes, natural killer cells, macrophages and dendritic cells, which are enhanced by increased expression or activation of Src kinases [42]. Hence, it can be suggested that the use of Src inhibitors in the treatment of cancers may inhibit host immune mechanisms against cancer cells and may render patients vulnerable to infections. Such adverse effects of Src inhibitors on host immunity has been shown for leukemia patients and emphasizes the need for use of Src inhibitors with caution [43].

## 4. Materials and Methods

### 4.1. Human Ethics Approval

This research project was approved by the Human Research and Ethics Committee (HREC approval #09/09) of The Royal Women’s Hospital, Melbourne, Australia. Human samples were obtained from patients undergoing treatment at The Royal Women’s Hospital after obtaining written consent under protocols approved by the Human Ethics approval #09/09.

### 4.2. Human Primary Tissue Collection and Preparation

Primary ovarian tissues were obtained from patients requiring surgery at the Oncology Dysplasia Unit of The Royal Women’s Hospital, Melbourne, Australia. The histopathological diagnosis, including tumour grade and stage were determined by independent pathologists at The Royal Women’s Hospital, Melbourne, Australia as part of the clinical diagnosis. Normal ovarian tissue samples were obtained from women undergoing total abdominal hysterectomy or bilateral salpingo-oophorectomy due to pre-diagnosed medical conditions. Benign tissue samples were obtained from patients who were presented with benign ovarian cysts and required laparotomy. Patients treated with chemotherapy, immunotherapy or radiation prior to surgery was omitted from this study. At the time of collection, tissue samples were fixed in 4% paraformaldehyde, or snap frozen in liquid nitrogen and stored at −80 °C.

### 4.3. Human Ascites Collection

Human ascites samples were collected from patients diagnosed with advanced-stage (FIGO stages III–IV) serous ovarian carcinoma, and adenocarcinoma Not Otherwise Specified (NOS). Samples were collected during debulking surgery and before the commencement of any chemotherapy treatment (termed chemonaïve), or at the time of disease recurrence by paracentesis where patients were not all treated identically but had previously received combinations of chemotherapies (termed chemotherapy treated). The samples were processed for the isolation of ascites-derived tumour cells as we have described previously [44]. Briefly, on the day of collection, ascetic fluid samples were centrifuged at 1200 rpm for 5 min at room temperature, and the supernatant was discarded. Contaminating red blood cells in the cell pellet of ascites were removed by hypotonic lysis and centrifuged at 1200 rpm for 5 min at room temperature. After discarding the supernatant, the bulk of the ascites cells were resuspended in a 1:1 ratio of MCDB 131 (Thermo Fisher, Scoresby, Australia) and DMEM (Sigma-Aldrich, St. Louis, MO, USA) supplemented with 10% (*v/v*) heat inactivated FBS (Thermo Fisher Scientific, Waltham, MA, USA), 2 mM L-glutamine (MCRI), and 1% (*v/v*) penicillin and streptomycin (MCRI), then seeded onto Costar® 6 well clear flat bottom ultra-low attachment multiwall plates or Corning® ultra-low attachment culture 100 mm petri dishes (Corning Incorporated, NY, USA). The ascites cells were incubated at 37 °C with 5% CO_2_ and grown as either non-adherent spheroid cells or adherent cells before collection within a week. Images of the collected ascites cells were captured using the Axiovert 100 phase contrast microscope (Zeiss, Oberkochen, Germany) and viewed using the DeltaPixView Software (https://www.deltapix.dk/software.html).

### 4.4. Cell Cultures

Ovarian serous carcinoma HEY cells (representative of Type II tumour) was obtained from Dr. Georgia Chenevix-Trench, Queensland Institute for Medical Research, Australia and has been described previously [18,28]. The cells were maintained in complete RPMI 1640 (Sigma-Aldrich, Sydney, Australia) supplemented with 10% (*v/v*) foetal bovine serum (Thermo Fisher Scientific), 2 mM L-glutamine (Murdoch Childrens Research Institute, Victoria, Australia) and 1% (*v/v*) penicillin and streptomycin (MCRI). Cells were maintained under standard culture conditions in 5% carbon dioxide infused incubators at 37 °C.

### 4.5. Drug Preparation

Paclitaxel (Hospira Australia, Melbourne, Victoria, Australia) was directly dissolved in respective complete growth medium to concentrations ranging from 0.0005–1 µg/mL. Dasatinib (Bristol Myers Squibb, New York, NY, USA) was dissolved in dimethyl sulfoxide (DMSO) ReagentPlus®, ≥99.5% (Sigma-Aldrich) and was either stored at −80 °C for 6 months, or dissolved in complete growth media for cell treatment to concentrations ranging from 1–50 µM. All drugs dissolved in complete growth media were prepared the day before and stored at 4 °C prior to treating cells.

### 4.6. Chemosensitivity Assay

Cells (2.5 × 10^4^) were seeded in triplicate in 96 well plates and incubated overnight under standard conditions. The following day, the media was discarded and replaced with various concentrations of paclitaxel [18], Dasatinib and combination treatments. After a 48 h incubation, the media was replaced with 100 μL of thiazolyl blue tetrazolium (MTT) solution (Sigma-Aldrich) dissolved in 1× PBS to a final concentration 0.5 mg/mL and incubated for 2 h at 37 °C with 5% CO_2_. The MTT reagent was then discarded and replaced with 100 μL of DMSO, and the plate was read at OD-595 nm using the SpectraMax190 Absorbance Microplate Reader and SoftMax® Pro Computer Software (https://www.moleculardevices.com). The average of each triplicate was expressed as the percentage of the average viable cells relative to untreated control cells.

### 4.7. Antibodies and Reagents

Polyclonal antibody against phosphorylated Src (p-Src) (Tyr416), total Src (t-Src), EpCAM and CD117 (c-Kit) were obtained from Cell Signalling Technology (Beverly, MA, USA). Monoclonal antibody against CD133 was obtained from Miltenyl Biotec (Macquarie Park, NSW, Australia). Antibodies against Ki67, CA125, OCT3/4 CD117 (c-Kit) and CD31 used for immunohistochemistry were obtained from Ventana (Roche, Basel, Switzerland).

### 4.8. Cell Preparation for Immunofluorescence

This was performed by the method described previously [18]. Viable cell numbers were determined using Trypan Blue. A concentration of 1 × 10^3^ for a cell line and 100–200 μL of non-adherent ascites-derived cells were seeded in an 8-well NuncTM Lab-Tek^TM^ Chamber Slide^TM^ System (Thermo Fisher Scientific) under standard conditions, until the cells reached 60% confluence. The growth medium was discarded, and replaced with fresh complete growth medium, or medium containing paclitaxel, Dasatinib or the combination. Cells were incubated again for a defined period as per experiment. Cells were fixed in 4% paraformaldehyde, and then blocked with CAS-Block^TM^ Histochemical Reagent (Thermo Fisher Scientific) for 10 min as per manufacturer’s instructions, followed by an overnight incubation at 4 °C with respective primary antibodies. After 2 washes with 1× PBS, cells were probed with Alexa Fluor® 488 conjugated Goat anti-Mouse antibody or Donkey anti-Goat, or Alexa Fluor® 568 conjugated Goat anti-Rabbit secondary antibodies at a 1:200 for 2 h at room temperature in the dark. 4’,6-diamidino-2-phenylindole (DAPI) (Life Technologies) was used to counter stained cells at a 1:50000 dilution for 5 min before mounting the slides with 80% glycerol and secured with coverslips.

Fluorescence imaging was visualized and captured using an Olympus CellR fluorescent microscope and associated software (Olympus Corporation, Tokyo, Japan). The CellR software has inbuilt analysis parameters that was set up at equal intensity all the time to avoid biased measurements. Semi-quantitative analysis was performed using the ImageJ software to assess the fluorescent intensity of individual staining per cell as published previously [18]. The results are expressed as a percentage of fold change of the protein of interest for each sample.

### 4.9. Western Blotting

This was performed as described previously [28]. After the cells were treated with paclitaxel, Dasatinib or the combination, protein was harvested (triplicates/group) using RIPA buffer supplemented with 2 mM sodium orthovanadate (Na_3_VO_4_), 2 mM phenylmethylsulfonyl fluoride (PMSF) and 1 mg/mL aprotonin (Sigma-Aldrich) and samples sonicated at an amplitude of 40 for 15 secs, using a Microson Ultrasonic cell disrupted (Misonic Incorporated, NY, USA). The extracted protein was quantified using the Quick StartTM Bradford Protein Assay Reagent Kit (Bio-Rad, Hercules, CA, USA). Samples were diluted to 40–50 μg in a 95:5 ratio of 2× Laemmli sample buffer (Bio-Rad) and β-mercaptoethanol (Sigma-Aldrich) and loaded into each well, with the pre-stained SDS-PAGE Precision Plus Protein^TM^ Kaleidoscope Standards (Bio-Rad) as a standard ladder. Electrophoresis was performed using a Mini-Protean II Electrophoresis Cell (Bio-Rad Laboratories) and transferred on to polyvinylidene fluoride or nitrocellulose membranes. The membranes were incubated in Super Signal® western blot Enhancer (Thermo Fisher Scientific) then blocked with 5% (*w/v*) BSA1 h at room temperature. Primary antibodies were applied for 48 h at 4 °C in the presence of SuperSignal® Diluent (Thermo Fisher Scientific), then probed with the secondary antibodies for 2 h at room temperature and visualised using SuperSignal^TM^ West Pico Chemiluminescent Substrate (Thermo Fisher Scientific) and a Fuji Film Las-4000 with associated software (GE Healthcare, Buckinghamshire, UK). Semi-quantitative densitometry analysis was performed using ImageJ software after standardisation to housekeeping GAPDH (Imgenex, San Diego, CA, USA).

### 4.10. Flow Cytometry

This was performed as we have described previously [18,45]. A cell pellet containing ~10^5^ cells per 200 μL was resuspended in 1× PBS and incubated with primary antibody with or without phycoerythrin (PE)-conjugated for 1 h at 4 °C. For non-PE-conjugated primary antibodies, cells were washed with 1× PBS, collected by centrifugation at 1200 rpm at 4 °C for 5 mins, and followed by two more washing process before incubating with PE-conjugated goat anti-mouse IgG monoclonal antibody (1:100) for 20–30 min at 4 °C. Cells were then washed with 1× PBS, centrifuged at 1200 rpm at 4 °C for 5 mins, and followed by two more washing process before the cell pellet was resuspended in 200 μL of 1× PBS. Cells were assayed using FACScan analytical flow cytometer (BD Biosciences, Franklin Lakes, NJ, USA) and data was analysed using the Cell Quest^TM^ software (BD Biosciences). The protein expression was measured using the Geo Mean value of the cell surface marker of interest relative to the IgG Geo Mean value used as a negative control. Results were expressed as the mean of cell fluorescent intensity shown as a histogram.

### 4.11. Mouse Experiments

Animal Ethics: This study was performed in strict accordance with the recommendations in the Guide for the Care and Use of the Laboratory Animals of the National Health and Medical Research Council of Australia. The experimental protocol was approved by the Department of Surgery, Royal Melbourne Hospital and University of Melbourne’s Animal Ethics Committee (Project-1413207.1).

### 4.12. Intraperitoneal Ovarian Cancer Model-Treatment Regimen

These experiments were performed as we have described previously [18,27]. Female Balb/c nu/nu mice (age 6–8 weeks) were obtained from the Animal Resources Centre, Western Australia and were housed in a standard pathogen-free environment with access to food and water. Viable cells (5 × 10^6^) were injected intraperitoneally (i.p) into each mouse. After 19 days, mice received weekly i.p injection of paclitaxel (15 mg/kg of mice body weight) and daily oral gavages of Dasatinib (10 mg/kg of mice bodyweight) (*n* = 5/group). Animals were euthanized at day 12 after starting treatment (Figure 5, Figure 6 and Figure 7). Organs (such as liver, kidney, gastrointestinal tract, pancreas and spleen) and solid tumours were collected and processed for H&E and/or immunohistochemical analysis. Endpoint criteria included loss of body weight exceeding 15% of initial body weight and general patterns of diminished wellbeing such as abnormalities in respiration, motility, posture, and response to provocation.

In another set of experiments, 19 days post-i.p injection with HEY cells, mice were divided into three groups based on the treatment given: paclitaxel, Dasatinib, and a combination of paclitaxel and Dasatinib (*n* = 5/group). All treatments were terminated when control mice reached the endpoint (as described above). Survival of individual mice was monitored with remaining animals until they reached endpoint (Figure 8).

### 4.13. Processing of Mice Tissues

Formalin-fixed mice organs and resected tumours were paraffin embedded by staff at the Anatomical Pathology Laboratory Services, The Royal Children’s Hospital, Melbourne, Australia. The tissues samples were dehydrated in 70% ethanol (2 h) and 90% ethanol (1 h) solutions before being immersed in xylene (2 h) and embedded in paraffin wax for 2 h.

### 4.14. Haematoxylin and Eosin Stain (H&E Stain)

H&E staining of mouse organs and tumours was performed by the staff at the Anatomical Pathology Laboratory Services located at The Royal Children’s Hospital, Melbourne, Australia according to the standard H&E protocol as described previously [28]. Paraffin embedded tissues were sectioned at 4 µm thickness, deparaffinised, rehydrated and stained for 3 min with Haematoxylin (Australian Biostain Pty Ltd, Traralgon, VIC, Australia). Slides were rinsed with 0.25% Acid Alcohol and Scott’s Tap water Substitute. Sections were stained with Eosin (Amber Scientific, Midvale, WA, Australia) for 2 min before final rinsing with absolute alcohol and xylene. The metastatic development was assessed by a qualified pathologist at The Royal Women’s Hospital (Melbourne, VIC, Australia). Histological images of stained organs and tumours were taken using Evos FL Auto 2 microscope (Thermo Fisher Scientific).

### 4.15. Immunohistochemical Analysis

Formalin-fixed, paraffin embedded 4 µm sectioned patient samples and mouse tumour xenografts were stained using a Ventana Benchmark Immunostainer (Ventana Medical Systems, Inc. Tucson, AZ, USA) as described previously [18,28]. Tumour sections were de-waxed with Ventana EZ Prep and endogenous peroxidise activity blocked using the Ventana Universal DAB inhibitor. Primary antibodies against p-Src, t-Src, Ki67, CA125, Oct4, CD117 (c-kit), CD133 and CD31 were diluted as per manufacturer’s instructions. The sections were counter-stained with Ventana Haematoxylin and Blueing solution, and primary antibody staining was detected using the ultra-View Universal DAB detection Kit (Roche, Basel, Switzerland). Negative controls were prepared by incubating each tumour sections without primary antibodies. Immunohistochemistry images were taken using an Evos FL Auto 2 microscope (Thermo Fisher Scientific). DAB staining (5× 200 µm images/section) was quantified using processing packages in ImageJ (positive pixels or number of positive cells) and Aperio (strong positive staining pixel count). Quantification was standardised to area and normalised to the negative control sections. Results displayed as mean ± SD.

### 4.16. Statistical Analysis

Welch’s t-test was used for the statistical analyses of all biological assays involving comparison between two groups. Comparisons between two groups with more than one independent variable were analysed using Two-Way ANOVA with Bonferroni’s post hoc test. Comparisons between more than two groups to designated control groups were analysed using One-Way ANOVA with Dunnett’s post hoc test. Comparisons between more than two groups with one independent variable were analysed using One-Way ANOVA with Tukey’s post hoc test. Comparisons of survival curves were analysed using Kaplan-Meier curves with Log-rank test. Each experiment was performed a minimum of three replicates using a minimum of three biological samples. Data was analysed using Microsoft Excel 2010 and GraphPad Prism Software (version 6).

## 5. Conclusions

In summary, we have shown that Src is significantly activated in high-stage ovarian tumours. Phosphorylated Src is also upregulated in response to chemotherapy treatment in ascites-derived patient’s tumour cells and in vitro ovarian cancer cells. As such, Src expression appears to correlate with chemoresistance and may contribute to subsequent recurrence and mortality in ovarian cancer patients, making it an appealing potential target in emerging therapeutic options. However, in this study, suppressing Src activation using multi-kinase inhibitor Dasatinib, only partially overcomes paclitaxel-induced effects in cells and tumours. Dasatinib appears to be limited in modulating paclitaxel-induced proliferative, tumourigenic, vasculogenic and CSC-mediated effects in ovarian cancer. This is supported by insignificant changes in Ki67, CA125, CD31, CD117 and OCT4 expression in combined paclitaxel and Dasatinib tumours, compared to paclitaxel-alone treated xenografts. In addition, Dasatinib lacks the ability to prolong disease-free progression in paclitaxel-induced tumours. This suggests that Src activation may not be the primary target in paclitaxel induced CSC-mediated chemoresistance and recurrence in ovarian cancer. A recent study has shown that simultaneous addition of Src inhibitors with paclitaxel may not be ideal for cancer control; however, sequential addition of Src inhibitor after paclitaxel treatment may overcome the adaptive paclitaxel-induced chemoresistance changes making resistant cells sensitive to dual therapy [46]. This approach, if reproducible, allows exploiting the adaptive changes induced in cancer cells by chemotherapy before they can be made susceptible to Src inhibitors. Studies like that warrant further investigation for future clinical trials on Src inhibitors.

## Figures and Tables

**Table 1:** Description of patients recruited for immunohistochemistry expression of Src activation.

Sample No.	Diagnosis	FIGO Stage	Age at Diagnosis	Genetic Background	Pre-Operative CA125	Ascites at Diagnosis	Survival
**Normal**							
1	Normal ovarian epithelium	-		BRCA+	-	No	-
2	Normal ovarian epithelium	-		Peutz-Jegher’s syndrome	-	No	-
**Benign**							
1	Benign cyst	-	19	-	-	No	-
2	Benign cyst	-	55	-	-	No	-
3	Large multi cysts ovarian mass	-	64	Family history of Ca	-	No	-
4	Benign cyst	-	58	Diagnosed with breast cancer	-	No	-
5	Large ovarian cyst	-	62	Unknown	-	No	-
6	Benign cyst	-	48	HNPCC carrier (colorectal cancer)	-	No	-
**Low-Stage**							
1	Serous cystadenocarcinoma NOS (Gr3)	Ic	90	Unknown	24	No	1 month ALC
2	Serous cystadenocarcinoma NOS	Ia	36	Unknown	138	No	2 years 4 months ALC
3	Serous cystadenocarcinoma (Gr1)	Ic	31	Unknown	177	No	4 years 10 months ALC
**High-Stage**							
1	Papillary serous adenocarcinoma (Gr3)		55	-	3025	Yes	2 years 5 months at death
2	Serous cystadenocarcinoma NOS (Gr2)	IIIc	65	Unknown	903	Yes	1 year 9 months at death
3	Carcinoma NOS (Gr3)	III	62	BRCA+	3058	Yes	2 years 7 months at death
4	Serous cystadenocarcinoma NOS (Gr3)	IIIc	38	Unknown	957	Yes	2 years 8 months ALC
5	Serous surface papillary carcinoma (Gr3)	IIIc	59	BRCA2+	397	Yes	4 years 7 months at death
6	Papillary serous adenocarcinoma (Gr2)	IIIc	41	Family history of ovarian cancer	Unknown	Unknown	8 years 8 months ALC
7	Serous cystadenocarcinoma NOS (Gr3)	IIIc	67	Unknown	Unknown	Yes	9 months at death
8	Papillary serous adenocarcinoma (Gr2)	IIIc	43	-	428	Yes	3 years 7 months at death
9	Papillary serous adenocarcinoma (Gr2)	IV	58	Family history of uterine cancer	3187	Yes	6 years 10 months at death
10	Papillary serous adenocarcinoma (Gr3)	IIIc	53	Unknown	1838	Yes	1 year 9 months at death

**Table 2:** Description of chemonaïve and recurrent patients recruited for the collection of ascites-derived tumour cells.

Sample Classification	Diagnosis	Grade	FIGO Stage	Treatment	Age at Diagnosis	Survival
Chemonaïve	Serous cystadenocarcinoma NOS	G3- poorly differentiated	IV	None	76	7 months at death
Chemonaïve	Serous cystadenocarcinoma NOS	G3- poorly differentiated	IIIc	None	64	1 month at death
Chemonaïve	Serous cystadenocarcinoma NOS	G3- poorly differentiated	IV	None	83	1 year 10 months at death
Chemonaïve	Serous cystadenocarcinoma NOS	G3- poorly differentiated	IIIc	None	41	1 year 7 months ALC
Chemonaïve	Serous cystadenocarcinoma NOS	G3- poorly differentiated	IIIc	None	52	1 year 9 months ALC
Chemonaïve	Carcinoma NOS	G3- poorly differentiated	IIc	None	62	2 years 2 months ALC
Chemonaïve	Serous cystadenocarcinoma NOS	G3- poorly differentiated	IIIc	None	63	9 months ALC
Chemonaïve	Adenocarcinoma NOS	G3- poorly differentiated	IV	None	52	1 year ALC
Recurrent	Serous cystadenocarcinoma NOS	G3- poorly differentiated	IV	Carboplatin and Paclitaxel 6 cycles Carboplatin 4 cycles Cisplatin 2 cycles Doxorubicin Pegylated Liposomal 1 cycle	50	1 year 10 months at death
Recurrent	Serous surface papillary carcinoma (Gr3)	G3- poorly differentiated	IIIc	ICON6 Trial 8 cycles Carboplatin and Paclitaxel 6 cycles OVAR16/VEG110655 Trial 4 cycles Cisplatin 6 cycles Doxorubicin Pegylated Liposomal 6 cycles Docetaxel 3 cycles REZOLVE Study 3 cycles Paclitaxel 1 cycl	59	4 years 7 months at death
Recurrent	Serous cystadenocarcinoma NOS (Gr3)	G3- poorly differentiated	IV	REZOLVE Study 5 cycles Unspecified Regimen 7 cycles Doxorubicin Pegylated Liposomal 6 cycles Paclitaxel 5 cycles Cisplatin 6 cycles	57	5 years 8 months at death
Recurrent	Serous cystadenocarcinoma NOS (Gr3)	G3- poorly differentiated	IIIc	Carboplatin and Paclitaxel 6 cycles PrePARE Study 2 cycles Cisplatin 5 cycles Unspecified Regimen 6 cycles Doxorubicin Pegylated Liposomal 5 cycles	61	2 years 2 months at death
Recurrent	Papillary serous adenocarcinoma (Gr2)	G1- well differentiated	IIIc	Carboplatin and Paclitaxel 3 cycles Doxorubicin Pegylated Liposomal 4 cycles	31	9 months at death
Recurrent	Papillary serous adenocarcinoma (Gr2)	G3- poorly differentiated	IIc	Carboplatin and Paclitaxel 6 cycles Lilly Trial 3 cycles Cisplatin 1 cycle Doxorubicin Pegylated Liposomal 3 cycles Paclitaxel 2 cycles REZOLVE Study 1 cycle	68	6 years 1 month at death

## Supplementary Materials

The following are available online at http://www.mdpi.com/2072-6694/11/2/243/s1. Figure S1: Effect of paclitaxel on the viability of ovarian cancer cell lines measured by MTT assay, Figure S2: Exposure of the TOV21G cells to paclitaxel enhances phosphorylation of Src in a time dependent manner, Figure S3: Effect of Dasatinib on the viability of ovarian cancer cell lines measured by MTT assay, Figure S4: Dasatinib inhibits paclitaxel-induced Src activation in TOV-21G cells, Figure S5: CSC-like marker expression is reduced with Dasatinib treatment, Figure S6: Single channel images relating to Figure 2 in the manuscript, Figure S7: Single channel images relating to Figure 3 in the manuscript.
