# Supplementary material for: Paclitaxel-Induced Src Activation Is Inhibited by Dasatinib Treatment, Independently of Cancer Stem Cell Properties, in a Mouse Model of Ovarian Cancer
Source: Cancers (Basel). 2019 Feb 19;11(2):243. doi: 10.3390/cancers11020243 (PMC6406511; doi:10.3390/cancers11020243)
Supplement: Supplementary file 1 [file cancers-11-00243-s001.pdf]

Supplementary Materials: Paclitaxel-Induced Src Activation is Inhibited by Dasatinib Treatment, Independently of Cancer Stem Cell Properties, in a Mouse Model of Ovarian Cancer

Elif Kadife, Emily Chan, Rodney Luwor, George Kannourakis, Jock Findlay and Nuzhat Ahmed

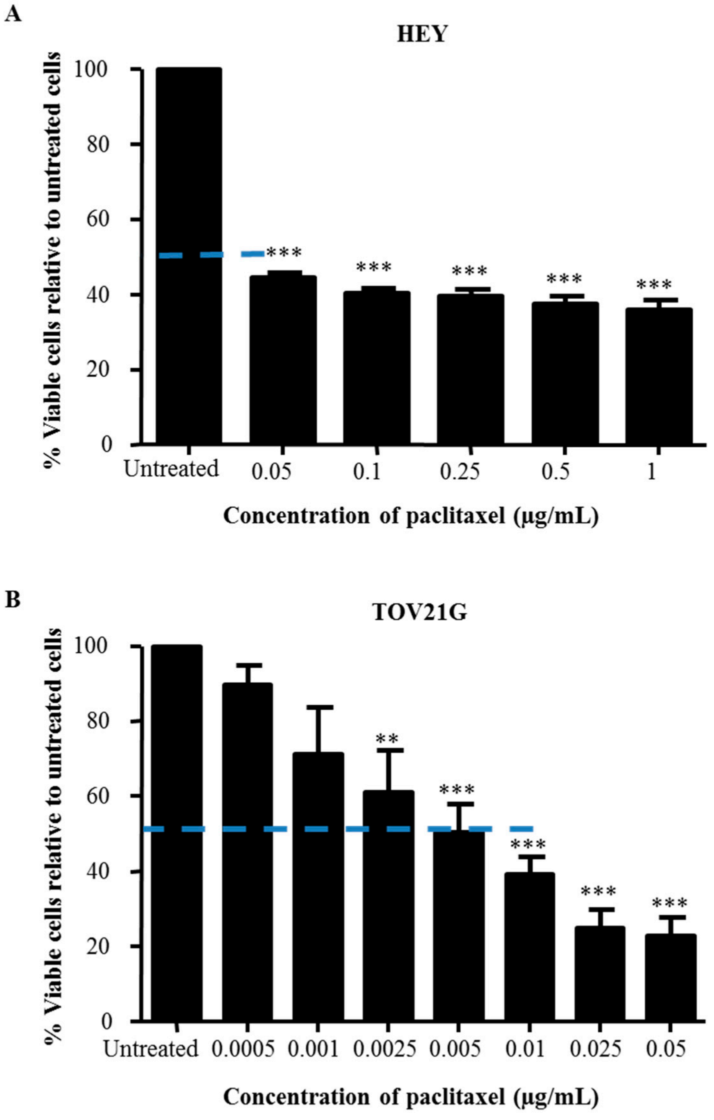

Figure S1. Effect of paclitaxel on the viability of ovarian cancer cell lines measured by MTT assay. Ovarian cancer (**A**) HEY and (**B**) TOV21G cell lines were treated with paclitaxel concentration ranging from 0.05–1 μg/mL and 0.0005–0.05 μg/mL respectively for 48 h before analysis by MTT assay. The graph represents four independent experiments performed in triplicate. Results are expressed as the percentage of the average OD reading relative to untreated cells ± SEM. Significance is indicated by ** $p < 0.01$, *** $p < 0.001$ compared to untreated. The intersected line in each graph represents the GI50.

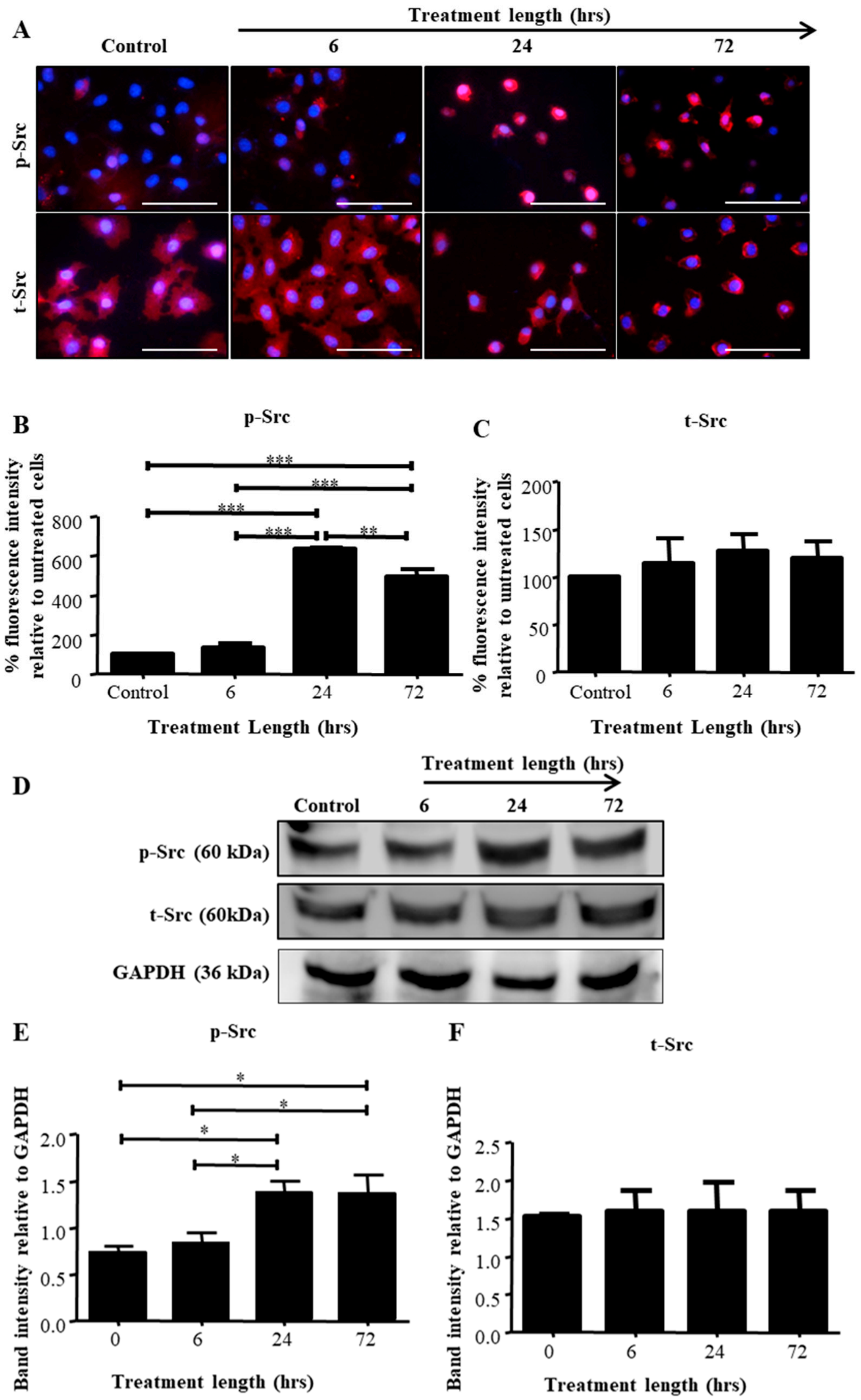

Figure 2. Exposure of the TOV21G cells to paclitaxel enhances phosphorylation of Src in a time dependent manner. (**A**) The expression of p-Src and t-Src was assessed by immunofluorescence in untreated and paclitaxel (0.01 µg/mL) treated cells following 6, 24 or 72 h of incubation. Staining was visualized using the secondary Alexa 590 (red) fluorescent-labelled antibody, and nuclei were detected by DAPI (blue) staining. Magnification 400× scale bar = 250 µm. Quantification of (**B**) p-Src and (**C**) t-Src fluorescent intensities was performed using Fiji software. Results are displayed as the

percentage of the average fluorescent intensity relative to untreated cells ± SEM (n = 3/group). (**D**) Total cell lysates of TOV21G cells were collected at 6, 24 and 72 h of paclitaxel treatment and were subjected to immunoblot analysis using antibodies specific for p- or t-Src or GAPDH. Images are representative of three independent experiments. Densitometric analysis of (**E**) p-Src and (**F**) t-Src protein expressions. The values represent the relative mean of band intensity normalized to GAPDH loading control ± SEM. Significance between the groups is indicated by * $p < 0.05$, ** $p < 0.01$, *** $p < 0.001$.

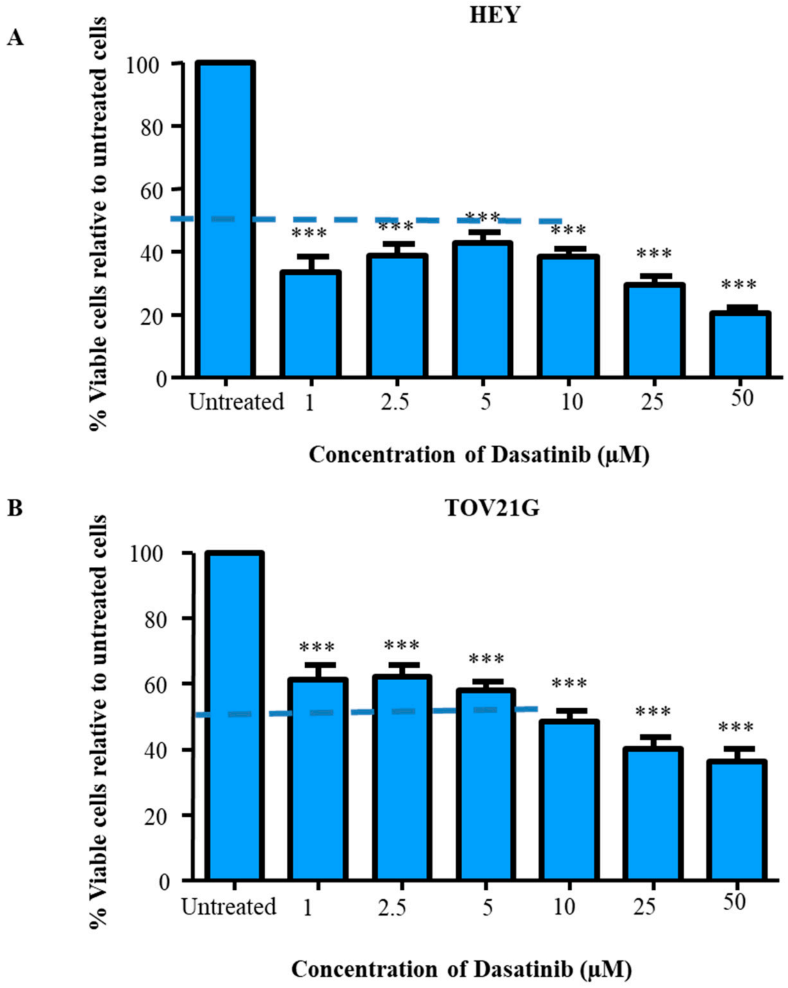

Figure S3. Effect of Dasatinib on the viability of ovarian cancer cell lines measured by MTT assay. Ovarian cancer (**A**) HEY and (**B**) TOV21G cell lines were treated with 1–50 µM of Dasatinib for 48 h before analysis by MTT assay. Results are expressed as the percentage of the average OD reading relative to untreated cells ± SEM of three independent experiments performed in triplicate. Significance is indicated by *** $p < 0.001$ compared to untreated. The intersected line in each graph represents the GI50.

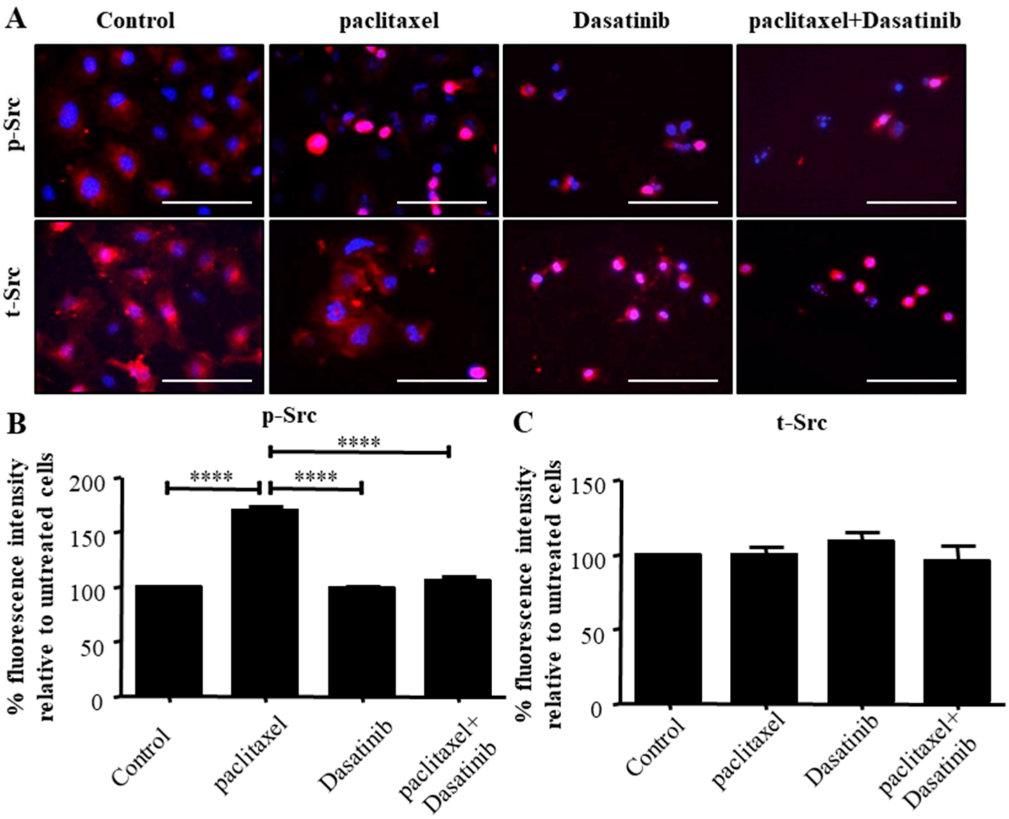

Figure S4. Dasatinib inhibits paclitaxel-induced Src activation in TOV-21G cells. (**A**) Immunofluorescent visualisation of **e**xpression and localization of the p- or t-Src proteins in untreated TOV21G cells or following a 24 h treatment with paclitaxel (0.01 μg/mL), Dasatinib (10 μM) or a combination of both. Staining was visualized using the secondary Alexa 590 (red) fluorescent-labelled antibodies, and nuclei were detected by DAPI (blue) staining. Images are representative of three independent experiments. Magnification 400× scale bar = 250 μm. Quantification of (**B**) p-Src and (**C**) t-Src fluorescent intensities was determined using Fiji software. Results are expressed as the percentage of the average fluorescent intensity value relative to untreated cells ± SEM (n = 3/group). Significance is indicated by **** $p < 0.0001$.

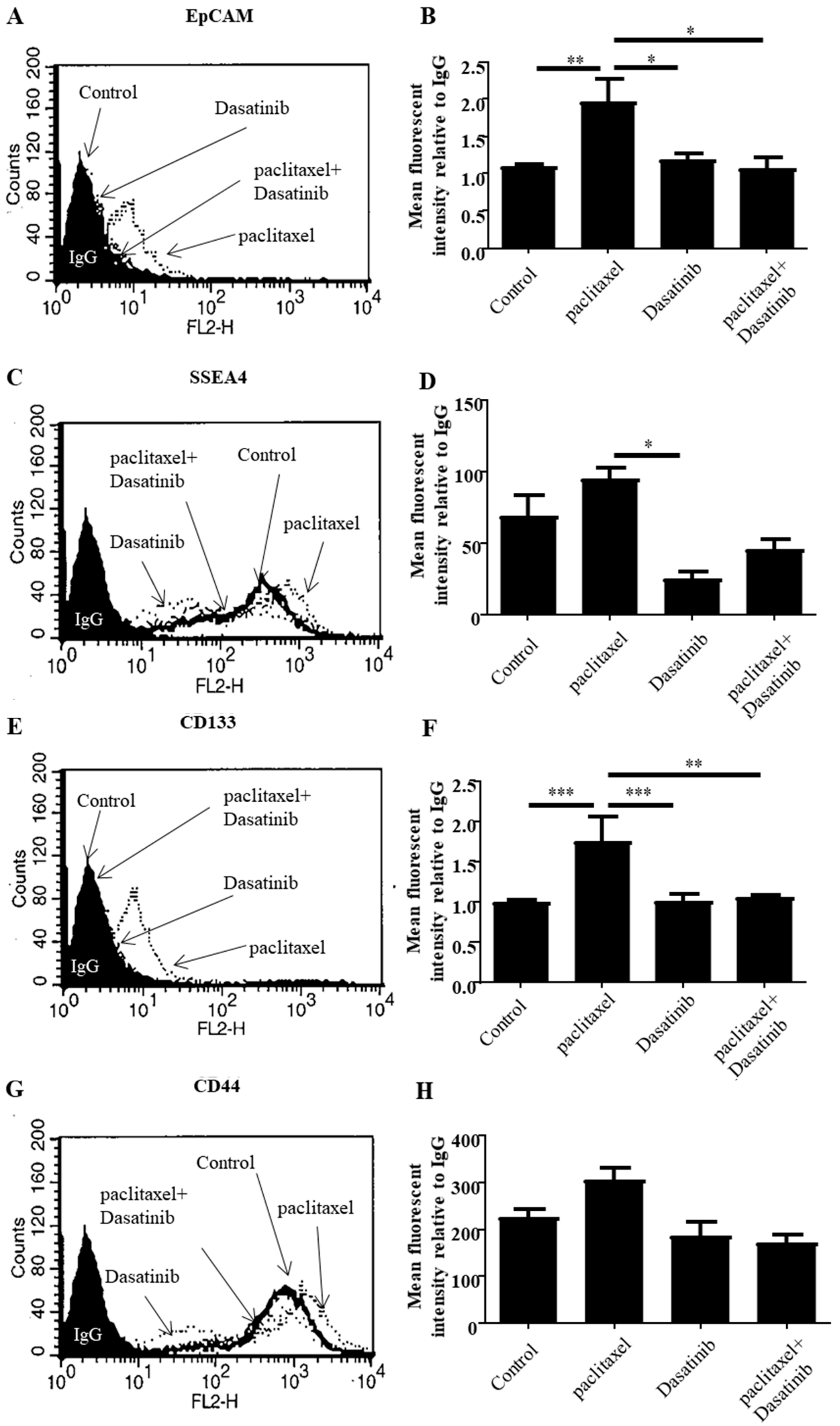

Figure S5. CSC-like marker expression is reduced with Dasatinib treatment in the TOV-21G cell line. Surface expression of (**A**,**B**) EpCAM, (**C**,**D**) SSEA-4, (**E**,**F**) CD133 and (**G**,**H**) CD44 in TOV-21G cell line following a 24 h treatment with paclitaxel (0.01 µg/mL), Dasatinib (10 µM) or a combination of both was deduced by Flow cytometry. Histograms are representative of four independent experiments. Semi-quantitative analysis of the arbitrary fluorescent expressions of (**B**) EpCAM, (**D**) SSEA-4, (**F**)

CD133 and (**H**) CD44 standardised to control IgG. Values represent the mean of fluorescent intensity relative to the mean of control IgG ± SEM ($n = 4$). Significance is indicated by * $p < 0.05$, ** $p < 0.01$, *** $p < 0.001$.

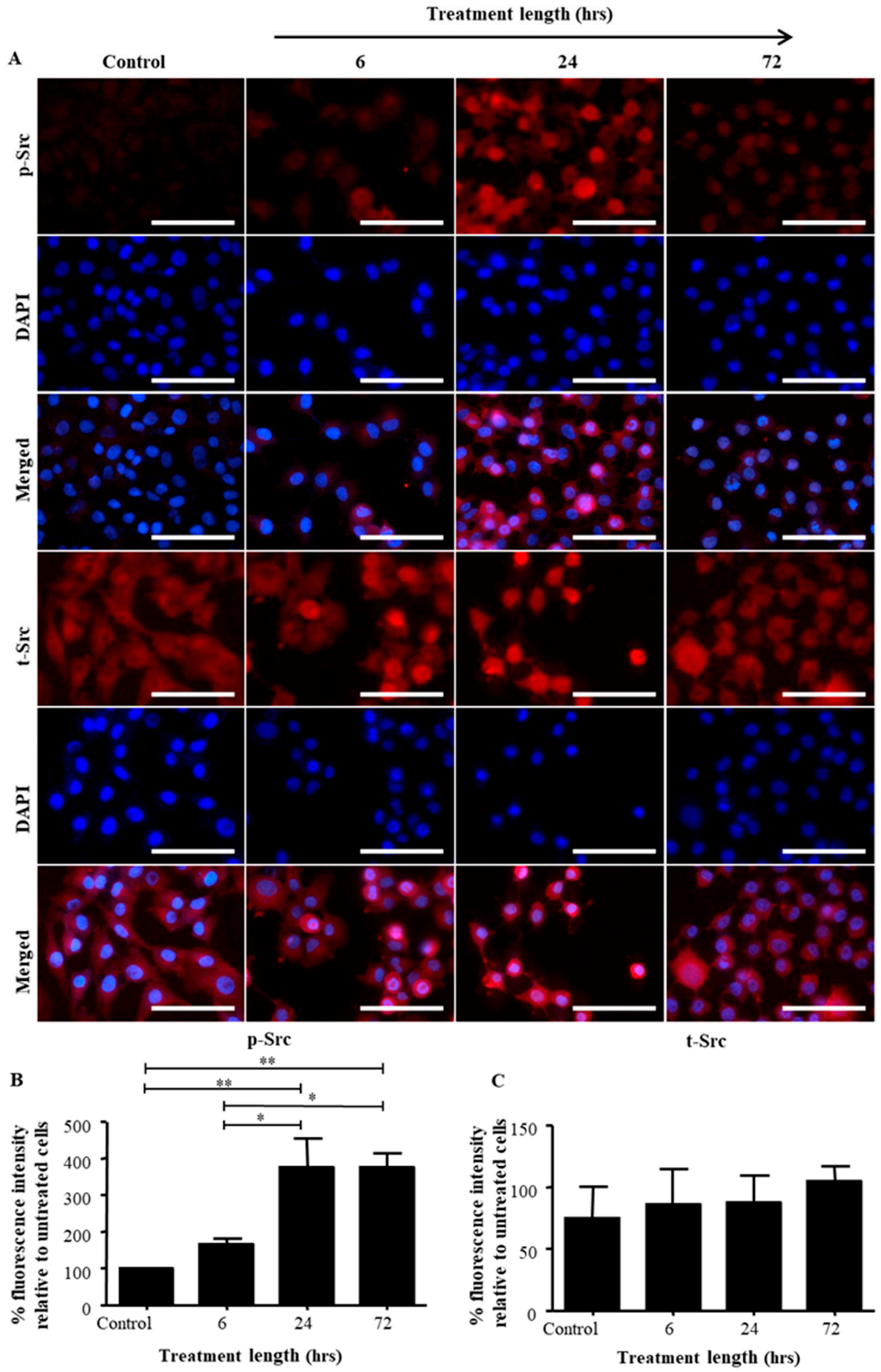

Figure S6. Single channel images relating to Figure 2 in the manuscript. (**A**) The expression of p-Src and t-Src was assessed by immunofluorescence in untreated and paclitaxel (0.05 µg/mL) treated HEY cells following 6, 24 or 72 h of incubation. Staining was visualized using the secondary Alexa 590 (red) fluorescent-labelled antibody, and nuclei were detected by DAPI (blue) staining. Magnification 400× scale bar = 250 µm. Quantification of (**B**) p-Src and (**C**) t-Src fluorescent intensities were performed using Fiji software. Results are expressed as the percentage of the average fluorescent intensity relative to untreated cells ± SEM ($n = 3$/group). Significance between the groups and is indicated by * $p < 0.05$, ** $p < 0.01$.

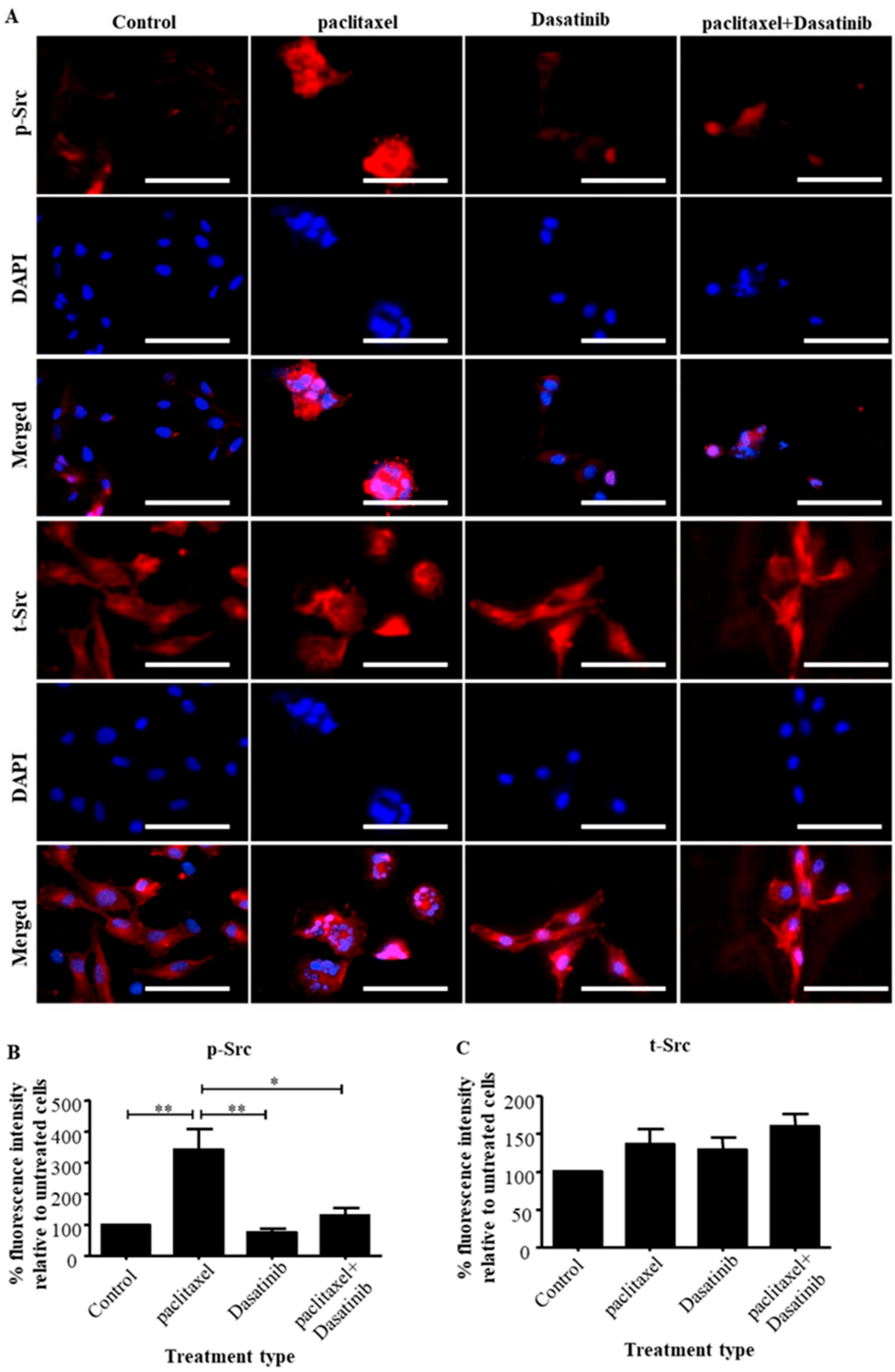

Figure S7. Single channel images relating to Figure 3 in the manuscript. (**A**) Expression and localization of the p- or t-Src in untreated, paclitaxel (0.05 μg/mL), Dasatinib (10 μM) or a combination of both treated HEY cells by immunofluorescence. Staining was visualized using the secondary Alexa 590 (red) fluorescent-labelled antibodies and nuclei were detected by DAPI (blue) staining. Images are representative of three independent experiments. Magnification 400× scale bar = 250 μm. Quantification of (**B**) p-Src and (**C**) t-Src fluorescent intensities were determined using Fiji software. Results are expressed as the percentage of the average fluorescent intensity value relative to untreated cells ± SEM (n = 3/group). Significance between the groups and is indicated by * $p < 0.05$, ** $p < 0.01$.

© 2019 by the authors. Licensee MDPI, Basel, Switzerland. This article is an open access article distributed under the terms and conditions of the Creative Commons Attribution (CC BY) license (http://creativecommons.org/licenses/by/4.0/).
